# 2,4,6-Trimethyl­pyridinium 4-nitro­benzoate–4-nitro­benzoic acid (1/1)

**DOI:** 10.1107/S1600536811032922

**Published:** 2011-08-27

**Authors:** Muhammad Athar Abbasi, Kenneth Nazir, Mehmet Akkurt, Aziz-ur-Rehman, Islam Ullah Khan, Ghulam Mustafa

**Affiliations:** a Department of Chemistry, Government College University, Lahore 54000, Pakistan; b Department of Physics, Faculty of Sciences, Erciyes University, 38039 Kayseri, Turkey

## Abstract

The asymmetric unit of the title co-crystal, C_8_H_12_N^+^·C_7_H_4_NO_4_^−^·C_7_H_5_NO_4_, contains two cations, two anions and two neutral 4-nitro­benzoic acid mol­ecules. In the crystal, O—H⋯O, N—H⋯O and C—H⋯O hydrogen bonds connect the ions and mol­ecules, forming a three-dimensional network.

## Related literature

For related structures, see: Ishida *et al.* (2004 ▶); Quah *et al.* (2008 ▶); Dong *et al.* (2010 ▶). For bond-length data, see: Allen *et al.* (1987) ▶.
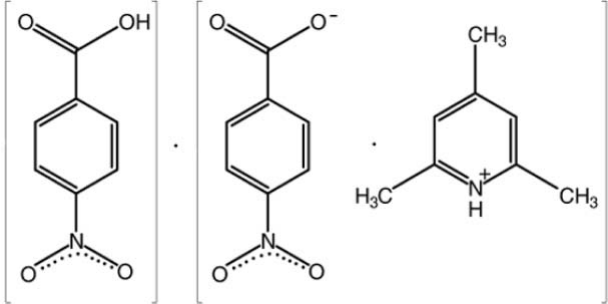


## Experimental

### 

#### Crystal data

C_8_H_12_N^+^·C_7_H_4_NO_4_^−^·C_7_H_5_NO_4_
*M* *_r_* = 455.42
Orthorhombic, 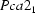

*a* = 14.4061 (15) Å
*b* = 8.4461 (10) Å
*c* = 36.787 (4) Å
*V* = 4476.1 (9) Å^3^
*Z* = 8
Mo *K*α radiation
μ = 0.11 mm^−1^
*T* = 296 K
0.54 × 0.44 × 0.37 mm

#### Data collection

Bruker APEXII CCD diffractometer
23612 measured reflections
5658 independent reflections
2131 reflections with *I* > 2σ(*I*)
*R*_int_ = 0.090

#### Refinement

*R*[*F* ^2^ > 2σ(*F* ^2^)] = 0.044
*wR*(*F* ^2^) = 0.102
*S* = 0.79
5658 reflections
602 parameters
1 restraint
H atoms treated by a mixture of independent and constrained refinement
Δρ_max_ = 0.18 e Å^−3^
Δρ_min_ = −0.17 e Å^−3^

### 

Data collection: *APEX2* (Bruker, 2007 ▶); cell refinement: *SAINT* (Bruker, 2007 ▶); data reduction: *SAINT*; program(s) used to solve structure: *SHELXS97* (Sheldrick, 2008 ▶); program(s) used to refine structure: *SHELXL97* (Sheldrick, 2008 ▶); molecular graphics: *ORTEP-3 for Windows* (Farrugia, 1997 ▶); software used to prepare material for publication: *WinGX* (Farrugia, 1999 ▶) and *PLATON* (Spek, 2009 ▶).

## Supplementary Material

Crystal structure: contains datablock(s) global, I. DOI: 10.1107/S1600536811032922/hb6339sup1.cif

Structure factors: contains datablock(s) I. DOI: 10.1107/S1600536811032922/hb6339Isup2.hkl

Supplementary material file. DOI: 10.1107/S1600536811032922/hb6339Isup3.cml

Additional supplementary materials:  crystallographic information; 3D view; checkCIF report

## Figures and Tables

**Table 1:** Hydrogen-bond geometry (Å, °)

*D*—H⋯*A*	*D*—H	H⋯*A*	*D*⋯*A*	*D*—H⋯*A*
N1*E*—H1*E*⋯O3*D*^i^	0.86	1.78	2.636 (4)	171
N1*F*—H1*F*⋯O4*C*^i^	0.86	1.76	2.613 (4)	173
O4*A*—H1*O*⋯O4*D*^ii^	0.90	1.67	2.549 (5)	164
O4*B*—H2*O*⋯O3*C*^iii^	0.90	1.65	2.536 (5)	165
C4*E*—H4*E*⋯O1*B*^iv^	0.93	2.58	3.398 (7)	147
C7*E*—H7*K*⋯O4*D*^v^	0.96	2.57	3.475 (6)	156
C7*E*—H7*L*⋯O1*B*^iv^	0.96	2.55	3.455 (9)	158

Symmetry codes: (i) 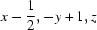; (ii) 
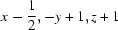
; (iii) 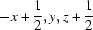; (iv) 
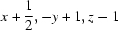
; (v) 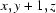.

**Fig. 1.**
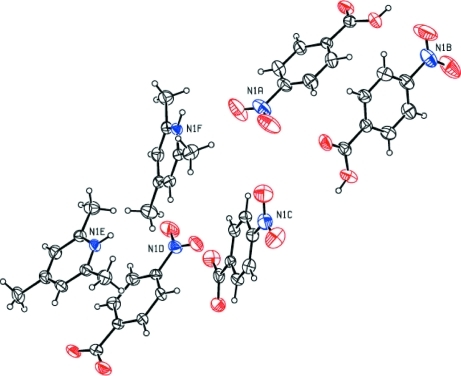
The molecules in the asymmetric unit of the title compound shown with 30% probability displacement ellipsoids. For clarity, only N atoms are labelled.

**Fig. 2.**
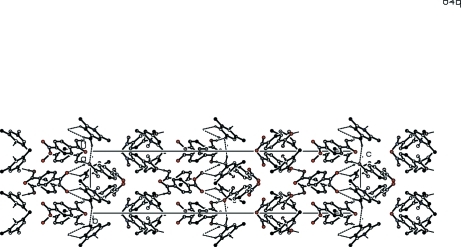
View of the packing and hydrogen bonding of the title compound down the a axis. H atoms not involved in hydrogen bonding have been omitted for clarity.

**Fig. 3.**
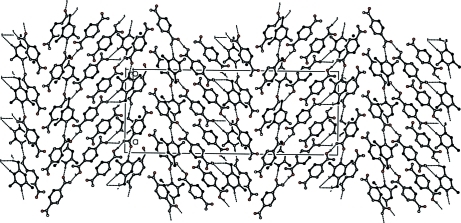
View of the packing and hydrogen bonding of the title compound down the b axis. H atoms not involved in hydrogen bonding have been omitted for clarity.

## supplementary crystallographic information

### Comment

The asymmetric unit of the title co-crystal, (I), contains two molecules of 2,4,6-trimethylpyridinium 4-nitrobenzoate and 4-nitrobenzoic acid (Fig. 1). All bond lengths and bond angles are as expected for this kind of compounds (Allen *et al.*, 1987).

In the structure, the molecular packing is stabilized by intermolecular O—H···O, N—H···O and C—H···O hydrogen bonds, forming a three dimensional network (Table 1, Figs. 2 & 3).

### Experimental

10 ml of ethanol were taken in 25 ml round bottom flask and 1.25 g of *p*-nitrobenzoic acid were added and suspension was heated to homogenize the mixture. Then 1 ml of *sym*-collidine (2,4,6-trimethylpyridine) was added and this mixture was refluxed for 1.5 h. Then this solution was concentrated on water bath to half amount and poured into sample vial and left for crystallization. On crystallization, colourless prisms of (I) were obtained.

### Refinement

The H atoms of the NH and OH groups in the title compound were placed in a difference map and refined with the distance restraint N—–H = 0.86 and O—–H = 0.90 Å; their *U*_iso_ values were constrained to be 1.2*U*_eq_ of the carrier atom. The remaining H atoms were positioned geometrically [C—H = 0.93 - 0.96 Å] and refined using a riding model, with*U*_iso_(H). = 1.2 *U*_eq_(C) and 1.5 *U*_eq_(C_methyl_). The structure was refined with Friedel pairs merged by the use of the MERG 4 instruction in *SHELXL97*, as the lack of anomalous scatterers did not allow the determination of the absolute configuration from the X-ray measurements. Two reflections 002 and 004 were omitted in the final refinement as these were obscured by beam stop.

### Crystal data

**Table:**

C_8_H_12_N^+^·C_7_H_4_NO_4_^−^·C_7_H_5_NO_4_	*F*(000) = 1904
*M**_r_* = 455.42	*D*_x_ = 1.352 Mg m^−^^3^
Orthorhombic, *P**c**a*2_1_	Mo *K*α radiation, λ = 0.71073 Å
Hall symbol: P 2c -2ac	Cell parameters from 2857 reflections
*a* = 14.4061 (15) Å	θ = 2.7–20.2°
*b* = 8.4461 (10) Å	µ = 0.11 mm^−^^1^
*c* = 36.787 (4) Å	*T* = 296 K
*V* = 4476.1 (9) Å^3^	Prism, colourless
*Z* = 8	0.54 × 0.44 × 0.37 mm

### Data collection

**Table:**

Bruker APEXII CCD diffractometer	2131 reflections with *I* > 2σ(*I*)
Radiation source: sealed tube	*R*_int_ = 0.090
graphite	θ_max_ = 28.3°, θ_min_ = 2.4°
φ and ω scans	*h* = −19→10
23612 measured reflections	*k* = −11→9
5658 independent reflections	*l* = −41→49

### Refinement

**Table:**

Refinement on *F*^2^	Secondary atom site location: difference Fourier map
Least-squares matrix: full	Hydrogen site location: inferred from neighbouring sites
*R*[*F*^2^ > 2σ(*F*^2^)] = 0.044	H atoms treated by a mixture of independent and constrained refinement
*wR*(*F*^2^) = 0.102	*w* = 1/[σ^2^(*F*_o_^2^) + (0.0372*P*)^2^] where *P* = (*F*_o_^2^ + 2*F*_c_^2^)/3
*S* = 0.79	(Δ/σ)_max_ < 0.001
5658 reflections	Δρ_max_ = 0.18 e Å^−^^3^
602 parameters	Δρ_min_ = −0.17 e Å^−^^3^
1 restraint	Extinction correction: *SHELXL97* (Sheldrick, 2008), FC^*^=KFC[1+0.001XFC^2^Λ^3^/SIN(2Θ)]^-1/4^
Primary atom site location: structure-invariant direct methods	Extinction coefficient: 0.0019 (3)

### Special details

**Table:**

Geometry. Bond distances, angles *etc*. have been calculated using the rounded fractional coordinates. All su's are estimated from the variances of the (full) variance-covariance matrix. The cell e.s.d.'s are taken into account in the estimation of distances, angles and torsion angles
Refinement. Refinement on *F*^2^ for ALL reflections except those flagged by the user for potential systematic errors. Weighted *R*-factors *wR* and all goodnesses of fit *S* are based on *F*^2^, conventional *R*-factors *R* are based on *F*, with *F* set to zero for negative *F*^2^. The observed criterion of *F*^2^ > σ(*F*^2^) is used only for calculating -*R*-factor-obs *etc*. and is not relevant to the choice of reflections for refinement. *R*-factors based on *F*^2^ are statistically about twice as large as those based on *F*, and *R*-factors based on ALL data will be even larger.

### Fractional atomic coordinates and isotropic or equivalent isotropic displacement parameters (Å^2^)

**Table:**

	*x*	*y*	*z*	*U*_iso_*/*U*_eq_	
N1E	0.0951 (2)	0.6583 (4)	0.01485 (10)	0.0507 (13)	
C1E	0.1321 (3)	0.7651 (5)	0.03852 (14)	0.0500 (19)	
C2E	0.2221 (3)	0.8086 (6)	0.03352 (15)	0.0610 (19)	
C3E	0.2737 (3)	0.7498 (6)	0.00536 (15)	0.0580 (18)	
C4E	0.2331 (3)	0.6421 (5)	−0.01785 (14)	0.0553 (16)	
C5E	0.1438 (3)	0.5961 (5)	−0.01280 (13)	0.0497 (16)	
C6E	0.0701 (3)	0.8265 (6)	0.06769 (13)	0.0703 (19)	
C7E	0.3732 (3)	0.7980 (6)	0.00030 (18)	0.089 (3)	
C8E	0.0923 (3)	0.4817 (6)	−0.03699 (16)	0.071 (2)	
N1F	−0.1353 (2)	0.8367 (4)	0.22390 (10)	0.0447 (13)	
C1F	−0.0849 (3)	0.8986 (5)	0.25124 (13)	0.0483 (16)	
C2F	0.0058 (3)	0.8497 (5)	0.25489 (13)	0.0560 (17)	
C3F	0.0433 (3)	0.7401 (6)	0.23086 (16)	0.0597 (19)	
C4F	−0.0131 (3)	0.6816 (5)	0.20373 (14)	0.0567 (19)	
C5F	−0.1040 (3)	0.7304 (5)	0.20001 (15)	0.0487 (19)	
C6F	−0.1295 (3)	1.0153 (6)	0.27559 (17)	0.072 (2)	
C7F	0.1435 (3)	0.6847 (7)	0.23554 (19)	0.096 (3)	
C8F	−0.1693 (3)	0.6715 (6)	0.17190 (15)	0.073 (2)	
O1A	0.3337 (3)	0.8459 (7)	0.82422 (15)	0.140 (3)	
O2A	0.4000 (3)	1.0348 (7)	0.85151 (18)	0.154 (3)	
O3A	0.0552 (2)	1.0121 (4)	0.98158 (12)	0.0765 (15)	
O4A	−0.01771 (19)	0.8303 (4)	0.94775 (9)	0.0700 (11)	
N1A	0.3371 (4)	0.9421 (8)	0.84779 (16)	0.098 (3)	
C1A	0.1158 (3)	0.8436 (6)	0.89404 (15)	0.064 (2)	
C2A	0.1873 (3)	0.8444 (6)	0.86886 (14)	0.0713 (19)	
C3A	0.2605 (3)	0.9417 (7)	0.87478 (14)	0.065 (2)	
C4A	0.2678 (3)	1.0374 (6)	0.90457 (16)	0.070 (2)	
C5A	0.1970 (3)	1.0351 (6)	0.92933 (16)	0.064 (2)	
C6A	0.1205 (3)	0.9379 (5)	0.92473 (13)	0.0490 (18)	
C7A	0.0485 (3)	0.9338 (6)	0.95380 (16)	0.0563 (19)	
O1B	−0.1107 (3)	0.3497 (8)	0.91296 (15)	0.150 (3)	
O2B	−0.1729 (4)	0.5467 (7)	0.88524 (17)	0.160 (3)	
O3B	0.1768 (2)	0.5131 (4)	0.75805 (11)	0.0763 (16)	
O4B	0.2464 (2)	0.3288 (4)	0.79171 (9)	0.0753 (11)	
N1B	−0.1128 (4)	0.4515 (8)	0.88884 (17)	0.097 (2)	
C1B	0.1109 (3)	0.3415 (6)	0.84378 (15)	0.066 (2)	
C2B	0.0394 (3)	0.3459 (6)	0.86889 (15)	0.075 (2)	
C3B	−0.0337 (3)	0.4471 (7)	0.86285 (16)	0.067 (2)	
C4B	−0.0389 (3)	0.5436 (6)	0.83350 (18)	0.077 (2)	
C5B	0.0330 (3)	0.5380 (6)	0.80844 (14)	0.0640 (19)	
C6B	0.1076 (3)	0.4385 (6)	0.81337 (15)	0.0530 (19)	
C7B	0.1806 (3)	0.4309 (7)	0.78477 (16)	0.060 (2)	
O1C	−0.2126 (3)	0.0101 (5)	0.11550 (15)	0.111 (2)	
O2C	−0.2654 (2)	0.2206 (6)	0.14139 (12)	0.109 (2)	
O3C	0.1291 (2)	0.2807 (5)	0.24426 (12)	0.0857 (16)	
O4C	0.19185 (19)	0.0829 (4)	0.21284 (11)	0.0787 (13)	
N1C	−0.2061 (3)	0.1190 (7)	0.13629 (14)	0.079 (2)	
C1C	0.0359 (3)	0.0609 (6)	0.16908 (14)	0.0567 (18)	
C2C	−0.0454 (3)	0.0446 (6)	0.14892 (13)	0.0603 (17)	
C3C	−0.1192 (3)	0.1379 (6)	0.15836 (14)	0.0550 (18)	
C4C	−0.1162 (3)	0.2450 (6)	0.18644 (15)	0.0570 (19)	
C5C	−0.0356 (3)	0.2575 (6)	0.20585 (14)	0.0513 (17)	
C6C	0.0404 (3)	0.1651 (5)	0.19758 (12)	0.0453 (16)	
C7C	0.1274 (3)	0.1787 (6)	0.22040 (16)	0.0597 (19)	
O1D	−0.0520 (2)	0.2967 (5)	0.08810 (10)	0.0843 (15)	
O2D	0.0105 (3)	0.4724 (6)	0.12218 (15)	0.116 (2)	
O3D	0.41943 (19)	0.4058 (4)	0.02846 (10)	0.0757 (13)	
O4D	0.3581 (2)	0.2083 (4)	−0.00369 (11)	0.0883 (18)	
N1D	0.0139 (3)	0.3771 (6)	0.09787 (13)	0.0653 (17)	
C1D	0.1914 (3)	0.2332 (5)	0.03318 (14)	0.0507 (19)	
C2D	0.1092 (3)	0.2513 (5)	0.05167 (14)	0.0550 (19)	
C3D	0.1047 (3)	0.3539 (5)	0.08009 (13)	0.0497 (17)	
C4D	0.1804 (3)	0.4396 (6)	0.09091 (13)	0.0587 (17)	
C5D	0.2617 (3)	0.4257 (5)	0.07166 (14)	0.0570 (19)	
C6D	0.2680 (2)	0.3227 (5)	0.04234 (13)	0.0473 (16)	
C7D	0.3560 (3)	0.3117 (6)	0.02039 (15)	0.0583 (19)	
H1E	0.03830	0.62960	0.01770	0.0610*	
H2E	0.24920	0.87990	0.04960	0.0730*	
H4E	0.26710	0.60070	−0.03710	0.0660*	
H6K	0.04390	0.73930	0.08090	0.1050*	
H6L	0.02120	0.88800	0.05700	0.1050*	
H6M	0.10540	0.89200	0.08400	0.1050*	
H7K	0.37990	0.90860	0.00570	0.1330*	
H7L	0.39150	0.77880	−0.02440	0.1330*	
H7M	0.41190	0.73730	0.01640	0.1330*	
H8K	0.02780	0.50980	−0.03770	0.1070*	
H8L	0.09880	0.37630	−0.02760	0.1070*	
H8M	0.11760	0.48630	−0.06110	0.1070*	
H1F	−0.19180	0.86790	0.22170	0.0530*	
H2F	0.04230	0.89040	0.27360	0.0670*	
H4F	0.01070	0.60740	0.18750	0.0680*	
H6P	−0.08390	1.05820	0.29190	0.1080*	
H6R	−0.17750	0.96420	0.28930	0.1080*	
H6S	−0.15590	1.09910	0.26130	0.1080*	
H7P	0.15710	0.60450	0.21780	0.1440*	
H7R	0.15160	0.64200	0.25950	0.1440*	
H7S	0.18480	0.77270	0.23220	0.1440*	
H8P	−0.19170	0.75890	0.15770	0.1100*	
H8R	−0.22070	0.61960	0.18350	0.1100*	
H8S	−0.13790	0.59780	0.15630	0.1100*	
H1A	0.06430	0.77930	0.89030	0.0770*	
H1O	−0.05570	0.82900	0.96720	0.0840*	
H2A	0.18510	0.77980	0.84840	0.0850*	
H4A	0.31930	1.10210	0.90790	0.0840*	
H5A	0.20040	1.10020	0.94970	0.0760*	
H1B	0.16100	0.27380	0.84730	0.0790*	
H2B	0.04070	0.28170	0.88940	0.0890*	
H2O	0.28270	0.31080	0.77220	0.0900*	
H4B	−0.08910	0.61140	0.83020	0.0920*	
H5B	0.03080	0.60260	0.78800	0.0770*	
H1C	0.08770	0.00040	0.16310	0.0680*	
H2C	−0.04940	−0.02690	0.12980	0.0730*	
H4C	−0.16740	0.30730	0.19210	0.0680*	
H5C	−0.03200	0.32940	0.22490	0.0610*	
H1D	0.19540	0.16000	0.01440	0.0610*	
H2D	0.05710	0.19380	0.04480	0.0660*	
H4D	0.17690	0.50620	0.11100	0.0700*	
H5D	0.31300	0.48590	0.07830	0.0680*	

### Atomic displacement parameters (Å^2^)

**Table:**

	*U*^11^	*U*^22^	*U*^33^	*U*^12^	*U*^13^	*U*^23^
N1E	0.0350 (17)	0.054 (2)	0.063 (3)	−0.0029 (18)	0.000 (2)	0.007 (2)
C1E	0.046 (3)	0.051 (3)	0.053 (4)	0.004 (2)	0.000 (3)	0.002 (3)
C2E	0.045 (3)	0.061 (3)	0.077 (4)	−0.006 (2)	−0.009 (3)	0.006 (3)
C3E	0.041 (2)	0.065 (3)	0.068 (4)	0.001 (2)	−0.001 (3)	0.015 (3)
C4E	0.041 (2)	0.066 (3)	0.059 (3)	0.007 (2)	0.010 (2)	0.011 (3)
C5E	0.047 (2)	0.055 (3)	0.047 (3)	0.006 (2)	0.006 (2)	0.002 (2)
C6E	0.074 (3)	0.075 (3)	0.062 (4)	0.012 (3)	0.006 (3)	−0.006 (3)
C7E	0.043 (3)	0.089 (4)	0.135 (6)	−0.015 (3)	0.002 (3)	0.027 (4)
C8E	0.067 (3)	0.067 (4)	0.079 (5)	−0.009 (3)	−0.003 (3)	−0.013 (3)
N1F	0.0351 (17)	0.050 (2)	0.049 (3)	−0.0017 (17)	−0.0008 (18)	0.004 (2)
C1F	0.047 (2)	0.048 (3)	0.050 (3)	−0.004 (2)	0.004 (2)	0.005 (2)
C2F	0.049 (3)	0.062 (3)	0.057 (3)	−0.008 (3)	−0.009 (2)	0.008 (3)
C3F	0.046 (3)	0.047 (3)	0.086 (4)	0.005 (2)	0.004 (3)	0.023 (3)
C4F	0.060 (3)	0.045 (3)	0.065 (4)	0.004 (2)	0.011 (3)	0.000 (3)
C5F	0.049 (3)	0.045 (3)	0.052 (4)	0.003 (2)	0.010 (2)	0.006 (3)
C6F	0.075 (3)	0.075 (4)	0.066 (4)	−0.005 (3)	0.010 (3)	−0.017 (3)
C7F	0.041 (3)	0.101 (4)	0.146 (6)	0.012 (3)	−0.009 (3)	0.020 (4)
C8F	0.075 (3)	0.068 (4)	0.076 (4)	−0.004 (3)	−0.024 (3)	−0.014 (3)
O1A	0.107 (3)	0.218 (6)	0.095 (4)	0.017 (4)	0.043 (3)	−0.028 (4)
O2A	0.093 (3)	0.212 (6)	0.157 (6)	−0.040 (4)	0.056 (3)	−0.017 (5)
O3A	0.0626 (19)	0.092 (3)	0.075 (3)	0.0027 (19)	0.017 (2)	−0.018 (2)
O4A	0.0501 (16)	0.091 (2)	0.069 (2)	−0.0086 (18)	0.0127 (17)	0.008 (2)
N1A	0.070 (3)	0.147 (6)	0.076 (4)	0.011 (3)	0.023 (3)	0.006 (4)
C1A	0.059 (3)	0.073 (4)	0.059 (4)	−0.015 (3)	−0.003 (3)	0.003 (3)
C2A	0.070 (3)	0.093 (4)	0.051 (3)	−0.004 (3)	0.011 (3)	−0.009 (3)
C3A	0.053 (3)	0.088 (4)	0.054 (4)	0.011 (3)	0.014 (3)	0.011 (3)
C4A	0.056 (3)	0.090 (4)	0.064 (4)	−0.013 (3)	0.007 (3)	0.001 (4)
C5A	0.058 (3)	0.068 (4)	0.065 (4)	−0.008 (3)	0.012 (3)	−0.005 (3)
C6A	0.041 (2)	0.053 (3)	0.053 (4)	0.006 (2)	0.005 (2)	0.006 (3)
C7A	0.041 (3)	0.058 (3)	0.070 (4)	0.005 (2)	−0.001 (3)	0.010 (3)
O1B	0.120 (4)	0.241 (7)	0.090 (4)	0.000 (4)	0.045 (3)	0.029 (4)
O2B	0.116 (4)	0.209 (6)	0.155 (5)	0.065 (4)	0.064 (4)	0.015 (5)
O3B	0.067 (2)	0.096 (3)	0.066 (3)	−0.007 (2)	0.021 (2)	0.018 (2)
O4B	0.0538 (18)	0.103 (2)	0.069 (2)	0.0109 (19)	0.0072 (17)	−0.021 (2)
N1B	0.081 (3)	0.140 (5)	0.070 (4)	−0.011 (3)	0.022 (3)	0.002 (4)
C1B	0.059 (3)	0.081 (4)	0.058 (4)	0.006 (3)	0.000 (3)	−0.007 (3)
C2B	0.076 (3)	0.094 (4)	0.054 (4)	−0.005 (3)	0.003 (3)	0.007 (3)
C3B	0.055 (3)	0.094 (4)	0.053 (4)	−0.007 (3)	0.005 (3)	−0.013 (3)
C4B	0.066 (3)	0.087 (4)	0.078 (5)	0.009 (3)	0.005 (3)	−0.002 (4)
C5B	0.063 (3)	0.070 (3)	0.059 (4)	0.005 (3)	0.013 (3)	0.007 (3)
C6B	0.049 (3)	0.059 (3)	0.051 (4)	−0.008 (2)	−0.001 (2)	−0.006 (3)
C7B	0.046 (3)	0.070 (4)	0.065 (4)	−0.010 (3)	0.004 (3)	−0.017 (3)
O1C	0.121 (3)	0.101 (4)	0.111 (4)	−0.011 (3)	−0.064 (3)	−0.004 (3)
O2C	0.067 (2)	0.128 (4)	0.131 (4)	0.016 (2)	−0.037 (2)	0.025 (3)
O3C	0.068 (2)	0.095 (3)	0.094 (3)	−0.011 (2)	−0.029 (2)	−0.025 (3)
O4C	0.0420 (17)	0.074 (2)	0.120 (3)	−0.0020 (18)	−0.0133 (19)	0.005 (2)
N1C	0.063 (3)	0.089 (4)	0.084 (4)	0.000 (3)	−0.013 (3)	0.028 (3)
C1C	0.040 (2)	0.061 (3)	0.069 (4)	0.002 (2)	0.003 (2)	0.003 (3)
C2C	0.070 (3)	0.060 (3)	0.051 (3)	−0.007 (3)	−0.007 (3)	0.003 (3)
C3C	0.041 (2)	0.059 (3)	0.065 (4)	−0.001 (2)	−0.015 (2)	0.015 (3)
C4C	0.048 (3)	0.054 (3)	0.069 (4)	0.009 (2)	0.006 (3)	0.010 (3)
C5C	0.048 (3)	0.054 (3)	0.052 (3)	−0.006 (2)	0.005 (3)	0.001 (3)
C6C	0.039 (2)	0.043 (3)	0.054 (3)	−0.005 (2)	−0.001 (2)	0.003 (2)
C7C	0.041 (3)	0.059 (3)	0.079 (4)	−0.013 (3)	−0.007 (3)	0.014 (3)
O1D	0.0528 (18)	0.106 (3)	0.094 (3)	0.001 (2)	0.019 (2)	0.023 (2)
O2D	0.115 (3)	0.130 (4)	0.102 (4)	0.011 (3)	0.053 (3)	−0.024 (3)
O3D	0.0381 (15)	0.082 (2)	0.107 (3)	0.0000 (17)	0.0182 (18)	−0.013 (2)
O4D	0.067 (2)	0.090 (3)	0.108 (4)	−0.003 (2)	0.035 (2)	−0.036 (3)
N1D	0.066 (3)	0.072 (3)	0.058 (3)	0.014 (2)	0.025 (2)	0.016 (3)
C1D	0.046 (3)	0.047 (3)	0.059 (4)	−0.003 (2)	0.012 (3)	−0.003 (3)
C2D	0.038 (3)	0.058 (3)	0.069 (4)	−0.005 (2)	0.012 (2)	0.006 (3)
C3D	0.048 (3)	0.052 (3)	0.049 (3)	0.013 (2)	0.013 (2)	0.015 (3)
C4D	0.061 (3)	0.060 (3)	0.055 (3)	0.003 (3)	0.008 (2)	−0.010 (3)
C5D	0.048 (3)	0.063 (3)	0.060 (4)	−0.001 (2)	−0.008 (3)	−0.005 (3)
C6D	0.038 (2)	0.048 (3)	0.056 (3)	0.008 (2)	0.007 (2)	0.007 (3)
C7D	0.042 (3)	0.057 (3)	0.076 (4)	0.006 (3)	0.006 (3)	0.000 (3)

### Geometric parameters (Å, °)

**Table:**

O1A—N1A	1.189 (9)	C4F—H4F	0.9300
O2A—N1A	1.205 (8)	C6F—H6P	0.9600
O3A—C7A	1.221 (7)	C6F—H6R	0.9600
O4A—C7A	1.313 (6)	C6F—H6S	0.9600
O4A—H1O	0.9000	C7F—H7S	0.9600
O1B—N1B	1.236 (9)	C7F—H7P	0.9600
O2B—N1B	1.189 (9)	C7F—H7R	0.9600
O3B—C7B	1.205 (7)	C8F—H8P	0.9600
O4B—C7B	1.307 (6)	C8F—H8S	0.9600
O4B—H2O	0.9000	C8F—H8R	0.9600
O1C—N1C	1.200 (7)	C1A—C6A	1.383 (7)
O2C—N1C	1.225 (7)	C1A—C2A	1.385 (7)
O3C—C7C	1.230 (7)	C2A—C3A	1.355 (7)
O4C—C7C	1.263 (6)	C3A—C4A	1.366 (8)
O1D—N1D	1.221 (6)	C4A—C5A	1.368 (7)
N1E—C1E	1.362 (6)	C5A—C6A	1.385 (6)
N1E—C5E	1.343 (6)	C6A—C7A	1.490 (7)
O2D—N1D	1.204 (7)	C1A—H1A	0.9300
O3D—C7D	1.247 (6)	C2A—H2A	0.9300
O4D—C7D	1.244 (6)	C4A—H4A	0.9300
N1E—H1E	0.8600	C5A—H5A	0.9300
N1F—C5F	1.335 (6)	C1B—C6B	1.387 (8)
N1F—C1F	1.346 (6)	C1B—C2B	1.384 (7)
N1F—H1F	0.8600	C2B—C3B	1.374 (7)
N1A—C3A	1.484 (7)	C3B—C4B	1.355 (8)
N1B—C3B	1.488 (8)	C4B—C5B	1.387 (7)
N1C—C3C	1.501 (6)	C5B—C6B	1.376 (7)
N1D—C3D	1.476 (6)	C6B—C7B	1.489 (7)
C1E—C2E	1.360 (6)	C1B—H1B	0.9300
C1E—C6E	1.489 (7)	C2B—H2B	0.9300
C2E—C3E	1.368 (7)	C4B—H4B	0.9300
C3E—C7E	1.502 (6)	C5B—H5B	0.9300
C3E—C4E	1.378 (7)	C1C—C2C	1.393 (6)
C4E—C5E	1.357 (6)	C1C—C6C	1.370 (7)
C5E—C8E	1.509 (7)	C2C—C3C	1.368 (7)
C2E—H2E	0.9300	C3C—C4C	1.374 (7)
C4E—H4E	0.9300	C4C—C5C	1.367 (7)
C6E—H6L	0.9600	C5C—C6C	1.379 (6)
C6E—H6K	0.9600	C6C—C7C	1.513 (7)
C6E—H6M	0.9600	C1C—H1C	0.9300
C7E—H7M	0.9600	C2C—H2C	0.9300
C7E—H7K	0.9600	C4C—H4C	0.9300
C7E—H7L	0.9600	C5C—H5C	0.9300
C8E—H8M	0.9600	C1D—C2D	1.374 (6)
C8E—H8L	0.9600	C1D—C6D	1.379 (6)
C8E—H8K	0.9600	C2D—C3D	1.360 (7)
C1F—C6F	1.479 (7)	C3D—C4D	1.368 (6)
C1F—C2F	1.377 (6)	C4D—C5D	1.374 (6)
C2F—C3F	1.389 (7)	C5D—C6D	1.389 (7)
C3F—C4F	1.379 (7)	C6D—C7D	1.506 (6)
C3F—C7F	1.527 (6)	C1D—H1D	0.9300
C4F—C5F	1.380 (6)	C2D—H2D	0.9300
C5F—C8F	1.484 (7)	C4D—H4D	0.9300
C2F—H2F	0.9300	C5D—H5D	0.9300
			
C7A—O4A—H1O	108.00	C5F—C8F—H8P	110.00
C7B—O4B—H2O	112.00	C2A—C1A—C6A	120.4 (4)
C1E—N1E—C5E	122.6 (3)	C1A—C2A—C3A	118.3 (5)
C1E—N1E—H1E	119.00	C2A—C3A—C4A	123.2 (5)
C5E—N1E—H1E	119.00	N1A—C3A—C4A	118.6 (5)
C1F—N1F—C5F	124.8 (3)	N1A—C3A—C2A	118.2 (5)
C5F—N1F—H1F	118.00	C3A—C4A—C5A	117.9 (4)
C1F—N1F—H1F	118.00	C4A—C5A—C6A	121.4 (5)
O2A—N1A—C3A	119.0 (6)	C5A—C6A—C7A	118.7 (4)
O1A—N1A—O2A	123.9 (6)	C1A—C6A—C7A	122.6 (4)
O1A—N1A—C3A	117.1 (6)	C1A—C6A—C5A	118.7 (4)
O2B—N1B—C3B	120.2 (6)	O3A—C7A—O4A	124.1 (5)
O1B—N1B—C3B	115.2 (5)	O4A—C7A—C6A	113.6 (4)
O1B—N1B—O2B	124.6 (6)	O3A—C7A—C6A	122.2 (4)
O1C—N1C—O2C	125.5 (5)	C2A—C1A—H1A	120.00
O2C—N1C—C3C	115.1 (5)	C6A—C1A—H1A	120.00
O1C—N1C—C3C	119.4 (5)	C3A—C2A—H2A	121.00
O1D—N1D—C3D	119.0 (4)	C1A—C2A—H2A	121.00
O2D—N1D—C3D	117.0 (4)	C3A—C4A—H4A	121.00
O1D—N1D—O2D	124.0 (5)	C5A—C4A—H4A	121.00
N1E—C1E—C2E	117.7 (4)	C6A—C5A—H5A	119.00
N1E—C1E—C6E	117.2 (4)	C4A—C5A—H5A	119.00
C2E—C1E—C6E	125.1 (4)	C2B—C1B—C6B	119.8 (4)
C1E—C2E—C3E	121.5 (5)	C1B—C2B—C3B	118.6 (5)
C2E—C3E—C4E	118.6 (4)	C2B—C3B—C4B	123.1 (5)
C2E—C3E—C7E	120.9 (5)	N1B—C3B—C4B	117.0 (5)
C4E—C3E—C7E	120.5 (5)	N1B—C3B—C2B	119.9 (5)
C3E—C4E—C5E	120.5 (4)	C3B—C4B—C5B	117.9 (4)
N1E—C5E—C8E	116.1 (4)	C4B—C5B—C6B	121.1 (5)
N1E—C5E—C4E	119.2 (4)	C5B—C6B—C7B	119.0 (5)
C4E—C5E—C8E	124.7 (4)	C1B—C6B—C7B	121.3 (4)
C1E—C2E—H2E	119.00	C1B—C6B—C5B	119.6 (4)
C3E—C2E—H2E	119.00	O4B—C7B—C6B	113.8 (5)
C5E—C4E—H4E	120.00	O3B—C7B—C6B	121.3 (4)
C3E—C4E—H4E	120.00	O3B—C7B—O4B	124.9 (5)
H6K—C6E—H6L	110.00	C2B—C1B—H1B	120.00
H6L—C6E—H6M	109.00	C6B—C1B—H1B	120.00
C1E—C6E—H6M	109.00	C3B—C2B—H2B	121.00
C1E—C6E—H6L	109.00	C1B—C2B—H2B	121.00
C1E—C6E—H6K	109.00	C3B—C4B—H4B	121.00
H6K—C6E—H6M	109.00	C5B—C4B—H4B	121.00
C3E—C7E—H7K	110.00	C6B—C5B—H5B	119.00
H7L—C7E—H7M	109.00	C4B—C5B—H5B	119.00
H7K—C7E—H7L	109.00	C2C—C1C—C6C	120.7 (4)
C3E—C7E—H7M	109.00	C1C—C2C—C3C	117.5 (5)
C3E—C7E—H7L	109.00	N1C—C3C—C2C	116.7 (5)
H7K—C7E—H7M	109.00	N1C—C3C—C4C	120.2 (4)
C5E—C8E—H8K	110.00	C2C—C3C—C4C	123.1 (4)
C5E—C8E—H8M	109.00	C3C—C4C—C5C	118.1 (4)
C5E—C8E—H8L	110.00	C4C—C5C—C6C	121.0 (5)
H8L—C8E—H8M	109.00	C5C—C6C—C7C	119.5 (4)
H8K—C8E—H8L	110.00	C1C—C6C—C7C	120.8 (4)
H8K—C8E—H8M	109.00	C1C—C6C—C5C	119.7 (4)
C2F—C1F—C6F	123.6 (4)	O3C—C7C—O4C	126.3 (5)
N1F—C1F—C2F	117.9 (4)	O3C—C7C—C6C	117.8 (4)
N1F—C1F—C6F	118.5 (4)	O4C—C7C—C6C	116.0 (5)
C1F—C2F—C3F	120.5 (4)	C2C—C1C—H1C	120.00
C2F—C3F—C7F	120.0 (5)	C6C—C1C—H1C	120.00
C4F—C3F—C7F	121.9 (5)	C3C—C2C—H2C	121.00
C2F—C3F—C4F	118.1 (4)	C1C—C2C—H2C	121.00
C3F—C4F—C5F	121.6 (4)	C3C—C4C—H4C	121.00
C4F—C5F—C8F	124.8 (4)	C5C—C4C—H4C	121.00
N1F—C5F—C8F	118.1 (4)	C4C—C5C—H5C	119.00
N1F—C5F—C4F	117.2 (4)	C6C—C5C—H5C	119.00
C3F—C2F—H2F	120.00	C2D—C1D—C6D	120.5 (4)
C1F—C2F—H2F	120.00	C1D—C2D—C3D	119.5 (4)
C5F—C4F—H4F	119.00	N1D—C3D—C2D	117.9 (4)
C3F—C4F—H4F	119.00	N1D—C3D—C4D	120.5 (4)
H6P—C6F—H6S	110.00	C2D—C3D—C4D	121.5 (4)
C1F—C6F—H6P	109.00	C3D—C4D—C5D	119.0 (4)
H6R—C6F—H6S	110.00	C4D—C5D—C6D	120.6 (4)
C1F—C6F—H6S	109.00	C1D—C6D—C5D	118.7 (4)
C1F—C6F—H6R	109.00	C1D—C6D—C7D	120.6 (4)
H6P—C6F—H6R	110.00	C5D—C6D—C7D	120.7 (4)
H7P—C7F—H7S	109.00	O3D—C7D—O4D	126.8 (4)
C3F—C7F—H7R	109.00	O3D—C7D—C6D	116.7 (4)
H7P—C7F—H7R	110.00	O4D—C7D—C6D	116.5 (4)
C3F—C7F—H7P	109.00	C2D—C1D—H1D	120.00
H7R—C7F—H7S	109.00	C6D—C1D—H1D	120.00
C3F—C7F—H7S	110.00	C1D—C2D—H2D	120.00
H8P—C8F—H8S	109.00	C3D—C2D—H2D	120.00
H8R—C8F—H8S	109.00	C3D—C4D—H4D	121.00
C5F—C8F—H8R	109.00	C5D—C4D—H4D	120.00
C5F—C8F—H8S	110.00	C4D—C5D—H5D	120.00
H8P—C8F—H8R	109.00	C6D—C5D—H5D	120.00
			
C5E—N1E—C1E—C2E	−0.2 (6)	C4A—C5A—C6A—C7A	176.4 (5)
C5E—N1E—C1E—C6E	179.2 (4)	C4A—C5A—C6A—C1A	−1.0 (7)
C1E—N1E—C5E—C4E	−0.8 (6)	C5A—C6A—C7A—O3A	−0.6 (7)
C1E—N1E—C5E—C8E	−178.9 (4)	C5A—C6A—C7A—O4A	−175.8 (4)
C5F—N1F—C1F—C2F	0.3 (7)	C1A—C6A—C7A—O3A	176.6 (5)
C1F—N1F—C5F—C8F	178.7 (4)	C1A—C6A—C7A—O4A	1.5 (7)
C5F—N1F—C1F—C6F	−179.9 (4)	C2B—C1B—C6B—C5B	−0.2 (8)
C1F—N1F—C5F—C4F	−0.3 (7)	C6B—C1B—C2B—C3B	0.2 (8)
O2A—N1A—C3A—C4A	−5.4 (9)	C2B—C1B—C6B—C7B	−176.7 (5)
O1A—N1A—C3A—C4A	172.2 (6)	C1B—C2B—C3B—C4B	−0.3 (8)
O1A—N1A—C3A—C2A	−6.8 (8)	C1B—C2B—C3B—N1B	178.4 (5)
O2A—N1A—C3A—C2A	175.6 (6)	N1B—C3B—C4B—C5B	−178.3 (5)
O1B—N1B—C3B—C4B	172.2 (6)	C2B—C3B—C4B—C5B	0.5 (8)
O2B—N1B—C3B—C4B	−6.7 (9)	C3B—C4B—C5B—C6B	−0.5 (8)
O1B—N1B—C3B—C2B	−6.6 (9)	C4B—C5B—C6B—C7B	176.9 (5)
O2B—N1B—C3B—C2B	174.5 (6)	C4B—C5B—C6B—C1B	0.3 (8)
O1C—N1C—C3C—C4C	−169.2 (5)	C1B—C6B—C7B—O3B	178.6 (5)
O1C—N1C—C3C—C2C	11.0 (8)	C5B—C6B—C7B—O3B	2.1 (8)
O2C—N1C—C3C—C4C	11.1 (7)	C1B—C6B—C7B—O4B	−1.3 (7)
O2C—N1C—C3C—C2C	−168.7 (5)	C5B—C6B—C7B—O4B	−177.8 (5)
O2D—N1D—C3D—C2D	−177.5 (5)	C6C—C1C—C2C—C3C	1.0 (7)
O1D—N1D—C3D—C2D	4.7 (7)	C2C—C1C—C6C—C5C	−1.6 (7)
O2D—N1D—C3D—C4D	−0.8 (7)	C2C—C1C—C6C—C7C	177.7 (5)
O1D—N1D—C3D—C4D	−178.6 (5)	C1C—C2C—C3C—C4C	0.0 (8)
C6E—C1E—C2E—C3E	−178.1 (5)	C1C—C2C—C3C—N1C	179.9 (5)
N1E—C1E—C2E—C3E	1.1 (7)	C2C—C3C—C4C—C5C	−0.4 (8)
C1E—C2E—C3E—C4E	−1.1 (8)	N1C—C3C—C4C—C5C	179.8 (5)
C1E—C2E—C3E—C7E	−179.6 (5)	C3C—C4C—C5C—C6C	−0.2 (8)
C2E—C3E—C4E—C5E	0.2 (7)	C4C—C5C—C6C—C7C	−178.1 (5)
C7E—C3E—C4E—C5E	178.6 (5)	C4C—C5C—C6C—C1C	1.1 (7)
C3E—C4E—C5E—C8E	178.7 (5)	C1C—C6C—C7C—O3C	175.9 (5)
C3E—C4E—C5E—N1E	0.8 (7)	C1C—C6C—C7C—O4C	−4.0 (7)
N1F—C1F—C2F—C3F	0.2 (7)	C5C—C6C—C7C—O3C	−4.8 (7)
C6F—C1F—C2F—C3F	−179.7 (5)	C5C—C6C—C7C—O4C	175.3 (5)
C1F—C2F—C3F—C7F	−179.0 (5)	C6D—C1D—C2D—C3D	−2.6 (7)
C1F—C2F—C3F—C4F	−0.6 (7)	C2D—C1D—C6D—C7D	−175.5 (4)
C2F—C3F—C4F—C5F	0.6 (7)	C2D—C1D—C6D—C5D	3.0 (7)
C7F—C3F—C4F—C5F	179.0 (5)	C1D—C2D—C3D—N1D	176.5 (4)
C3F—C4F—C5F—C8F	−179.1 (5)	C1D—C2D—C3D—C4D	−0.3 (7)
C3F—C4F—C5F—N1F	−0.2 (7)	N1D—C3D—C4D—C5D	−174.1 (4)
C2A—C1A—C6A—C7A	−176.0 (5)	C2D—C3D—C4D—C5D	2.5 (7)
C6A—C1A—C2A—C3A	−1.2 (7)	C3D—C4D—C5D—C6D	−2.0 (7)
C2A—C1A—C6A—C5A	1.3 (7)	C4D—C5D—C6D—C1D	−0.8 (7)
C1A—C2A—C3A—N1A	179.9 (5)	C4D—C5D—C6D—C7D	177.8 (4)
C1A—C2A—C3A—C4A	0.9 (8)	C1D—C6D—C7D—O3D	174.1 (4)
N1A—C3A—C4A—C5A	−179.6 (5)	C1D—C6D—C7D—O4D	−6.6 (7)
C2A—C3A—C4A—C5A	−0.6 (8)	C5D—C6D—C7D—O3D	−4.4 (7)
C3A—C4A—C5A—C6A	0.7 (8)	C5D—C6D—C7D—O4D	174.9 (4)

### Hydrogen-bond geometry (Å, °)

**Table:**

*D*—H···*A*	*D*—H	H···*A*	*D*···*A*	*D*—H···*A*
N1E—H1E···O3D^i^	0.86	1.78	2.636 (4)	171
N1F—H1F···O4C^i^	0.86	1.76	2.613 (4)	173
O4A—H1O···O4D^ii^	0.90	1.67	2.549 (5)	164
O4B—H2O···O3C^iii^	0.90	1.65	2.536 (5)	165
C4E—H4E···O1B^iv^	0.93	2.58	3.398 (7)	147
C7E—H7K···O4D^v^	0.96	2.57	3.475 (6)	156
C7E—H7L···O1B^iv^	0.96	2.55	3.455 (9)	158

Symmetry codes: (i) *x*−1/2, −*y*+1, *z*; (ii) *x*−1/2, −*y*+1, *z*+1; (iii) −*x*+1/2, *y*, *z*+1/2; (iv) *x*+1/2, −*y*+1, *z*−1; (v) *x*, *y*+1, *z*.
